# Beyond Pneumonia: A Case of Steroid-Responsive Pulmonary Eosinophilia With Atypical p-ANCA Positivity

**DOI:** 10.7759/cureus.116432

**Published:** 2026-09-17

**Authors:** Helena Awada, Samuel Tincher, Shehan M Thangaratnam, Sarkis Arabian

**Affiliations:** 1 Internal Medicine, Arrowhead Regional Medical Center, Colton, USA; 2 Internal Medicine, California University of Science and Medicine, Colton, USA; 3 Pulmonary and Critical Care, Arrowhead Regional Medical Center, Colton, USA; 4 Internal Medicine, Martin Luther King Community Health, Los Angeles, USA; 5 Critical Care, Arrowhead Regional Medical Center, Colton, USA

**Keywords:** anca-associated vasculitis, atypical p-anca, bronchoalveolar lavage, corticosteroid response, eosinophilic lung disease, ground-glass opacities, non-resolving pneumonia, organizing pneumonia, pulmonary eosinophilia, pulmonary vasculitis

## Abstract

Persistent pulmonary opacities despite antimicrobial therapy should prompt consideration of diagnoses beyond infection. We present a 76-year-old man with heart failure with moderately reduced ejection fraction, coronary artery disease, type 2 diabetes mellitus, and eczema treated with dupilumab who presented with progressive dyspnea, dry cough, weakness, hypoxemia, and a recent episode of hemoptysis. He had recently been hospitalized for presumed pneumonia and underwent bronchoscopy at an outside hospital before being discharged on amoxicillin, without significant improvement. Chest computed tomography (CT) demonstrated diffuse bilateral ground-glass opacities (GGOs), and he was again treated empirically for multifocal pneumonia. His symptoms and oxygen requirement persisted despite antibiotics, and an extensive infectious evaluation was unrevealing. Repeat CT showed persistent bilateral GGOs with increasing left basilar consolidation. Bronchoscopy with bronchoalveolar lavage (BAL) demonstrated mixed inflammation with 13% eosinophils, and cytology showed acute inflammation with eosinophils. Although several organisms were detected in BAL specimens, Infectious Disease considered these findings to represent colonization, contamination, or viral reactivation rather than active infection. Peripheral eosinophilia was present, with a peak absolute eosinophil count of 1.695 × 10³/µL and a peak eosinophil percentage of 15%. Indirect immunofluorescence demonstrated repeatedly positive atypical perinuclear antineutrophil cytoplasmic antibody (p-ANCA), with titers increasing from 1:40 to 1:80, while myeloperoxidase (MPO) and proteinase 3 (PR3) antibodies remained negative. Organizing pneumonia, eosinophilic lung disease, medication-associated pulmonary inflammation, and ANCA-associated vasculitis, including eosinophilic granulomatosis with polyangiitis, were considered. Prednisone 60 mg orally daily was initiated for the suspected inflammatory pulmonary process. The absolute eosinophil count decreased from 0.79 × 10³/µL (10%) immediately before corticosteroid initiation to 0.00 × 10³/µL (0%) the following day, and the patient was documented on room air approximately 48 hours after corticosteroid initiation. However, concurrent heart-failure-directed therapy and possible cardiogenic pulmonary edema limited attribution of the improvement solely to corticosteroids. This case illustrates the diagnostic challenge of non-resolving pulmonary infiltrates when eosinophilic inflammation, abnormal but non-specific ANCA testing, medication exposure, infection, and cardiogenic pulmonary edema provide competing explanations.

## Introduction

Ground-glass opacities (GGOs) are a common but non-specific finding on chest computed tomography (CT) and may occur with infection, pulmonary edema, inflammatory lung disease, autoimmune disease, and drug-induced lung injury. In a patient presenting with fever, dyspnea, hypoxemia, and bilateral infiltrates, pneumonia is often an appropriate initial diagnosis. Failure to improve despite adequate antimicrobial therapy, however, should prompt reconsideration of the differential diagnosis.

Organizing pneumonia (OP) is an important mimic of non-resolving pneumonia. It may be cryptogenic or occur secondary to infection, medications, autoimmune disease, malignancy, radiation, or other pulmonary injury [1,2]. Patients often present with subacute cough, dyspnea, constitutional symptoms, hypoxemia, and persistent pulmonary opacities [1,2]. Corticosteroids frequently result in clinical and radiographic improvement, although steroid responsiveness is not specific to OP.

Pulmonary eosinophilia provides another diagnostic clue. Eosinophils normally account for less than 1% of cells recovered by bronchoalveolar lavage (BAL) [3]. Marked BAL eosinophilia can support eosinophilic pneumonia, whereas more modest elevations, such as the 13% observed in this case, may indicate eosinophilic inflammation without establishing a specific eosinophilic pneumonia syndrome [3]. BAL eosinophilia therefore needs to be interpreted alongside peripheral eosinophilia, medication and exposure history, imaging, and extrapulmonary manifestations.

The combination of pulmonary infiltrates, eosinophilia, and neurologic or other systemic findings may also raise concern for eosinophilic granulomatosis with polyangiitis (EGPA). EGPA is a small- to medium-vessel vasculitis characterized by eosinophilic inflammation and is classically associated with asthma, sinonasal disease, peripheral eosinophilia, and variable systemic involvement. The 2022 American College of Rheumatology/European Alliance of Associations for Rheumatology (ACR/EULAR) criteria incorporate eosinophilia, obstructive airway disease, nasal polyposis, eosinophilic-predominant extravascular inflammation, and neuropathy [4]. These are classification criteria, however, and should not be used in isolation to establish the diagnosis.

Antineutrophil cytoplasmic antibody (ANCA) testing can support the diagnosis of ANCA-associated vasculitis (AAV), but the antigenic specificity of the antibody is important. Current recommendations emphasize antigen-specific testing for myeloperoxidase (MPO)-ANCA and proteinase 3 (PR3)-ANCA rather than reliance on immunofluorescence patterns alone [5]. An atypical perinuclear antineutrophil cytoplasmic antibody (p-ANCA) pattern in the absence of MPO or PR3 antibodies is less specific for AAV and may occur in other inflammatory disorders.

We describe an older man with persistent hypoxic respiratory failure and bilateral GGOs initially treated as multifocal pneumonia. BAL and peripheral eosinophilia, elevated IgE, neuropathic symptoms that reportedly worsened during the pulmonary illness, atypical p-ANCA positivity, and dupilumab exposure broadened the differential to include eosinophilic lung disease, medication-associated pulmonary inflammation, and pulmonary manifestations of systemic vasculitis. At the same time, heart failure with possible cardiogenic pulmonary edema remained an important competing explanation for the radiographic abnormalities. This case highlights the diagnostic challenge of interpreting eosinophilic inflammation and non-specific ANCA positivity when medication exposure, infection, cardiogenic pulmonary edema, and inflammatory lung disease remain plausible and potentially overlapping explanations.

## Case presentation

A 76-year-old man with a medical history of hypertension, coronary artery disease status post percutaneous coronary intervention with two stents, heart failure with moderately reduced ejection fraction (HFmrEF) of approximately 45%, type 2 diabetes mellitus with a hemoglobin A1c of 6.4%, chronic polyneuropathy, anemia of chronic disease, and eczema presented to the emergency department on HD 1 with approximately 10-14 days of progressive generalized weakness, fatigue, dry cough, shortness of breath, and dizziness. He had no documented history of asthma or inhaler use, and no wheezing was documented during the hospitalization.

Prior to presentation, the patient reported a fever as high as 103°F and had recently been hospitalized at an outside facility for presumed pneumonia, where he underwent bronchoscopy. He was subsequently discharged home on amoxicillin; however, his respiratory and constitutional symptoms failed to meaningfully improve. He also reported hemoptysis during his initial presentation, which resolved after several days. Despite the reported fever prior to admission, he was afebrile on arrival.

Initial chest radiography demonstrated cardiomegaly with multifocal bilateral pulmonary infiltrates. CT pulmonary angiography performed on HD 2 demonstrated diffuse bilateral GGOs without evidence of pulmonary embolism, pleural effusion, or right heart strain (Figure 1A). Laboratory evaluation was notable for hyponatremia with a serum sodium of approximately 129 mmol/L (reference range: 135-145 mmol/L). There was no significant leukocytosis, and serum procalcitonin was low. He required approximately 2 L/min of supplemental oxygen by nasal cannula. Given his recent fever, hypoxemia, bilateral infiltrates, and recent hospitalization for pneumonia, he was initially treated empirically for multifocal pneumonia with cefepime and azithromycin from HD 2 through HD 4. Blood cultures remained negative, and there was no meaningful clinical improvement with antimicrobial therapy.

**Figure 1:**
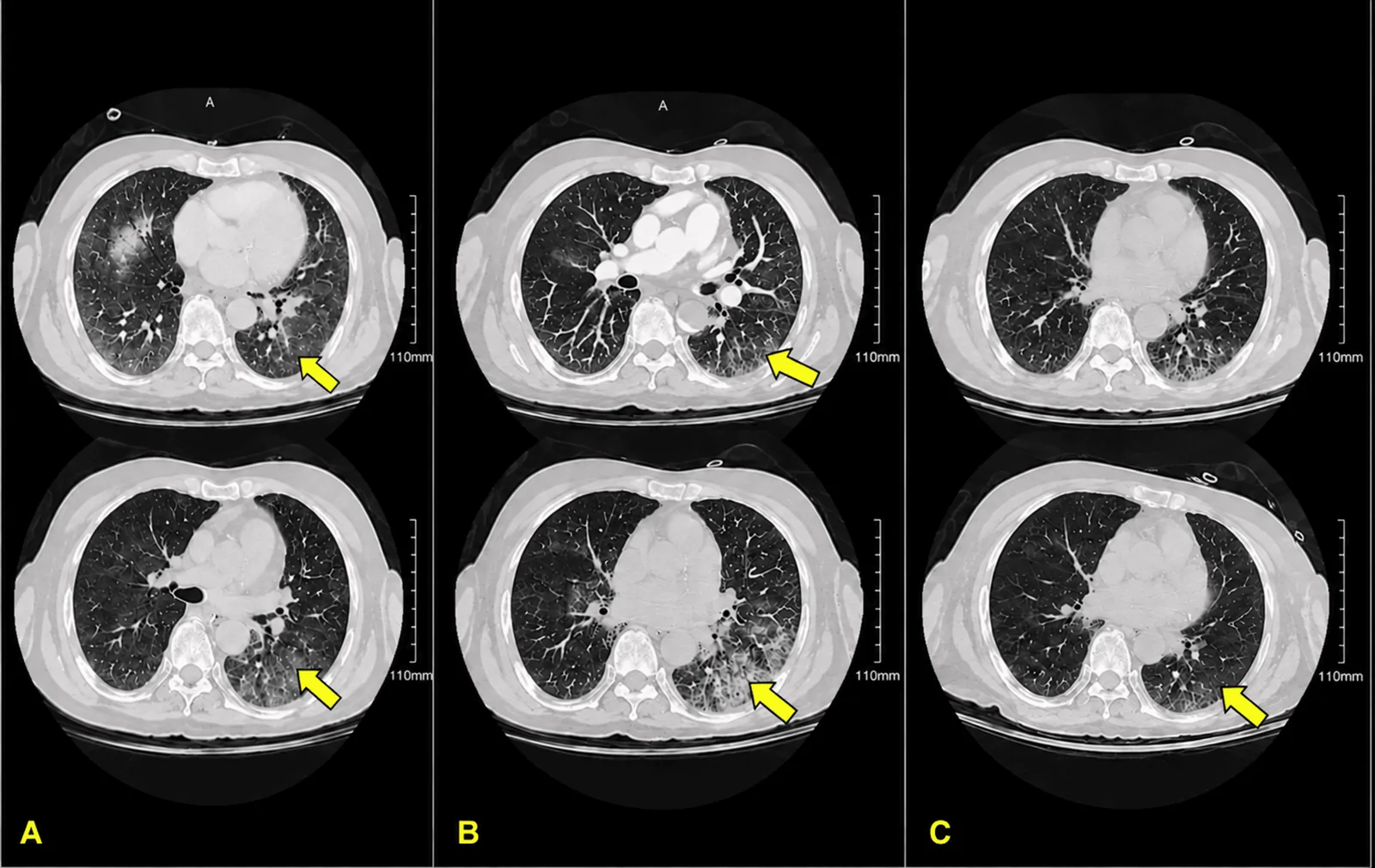
Serial chest computed tomography (CT) imaging demonstrating evolution of bilateral ground-glass opacities. (A) Initial CT on HD 2 demonstrating diffuse bilateral ground-glass opacities (GGOs). (B) Repeat CT on HD 12 demonstrating persistent basilar-predominant bilateral GGOs with increased consolidative opacity at the left lung base despite antimicrobial therapy. (C) Follow-up CT on HD 20 demonstrating interval improvement in the basilar-predominant GGOs and interlobular septal thickening, with new right lower lobe tree-in-bud nodular opacities. Cardiogenic pulmonary edema was considered a potential contributor to the radiographic abnormalities and subsequent improvement. Yellow arrows indicate representative areas of the pulmonary parenchymal abnormalities described in each panel. GGOs: ground-glass opacities; CT: computed tomography.

Despite broad-spectrum antimicrobial therapy, the patient's dry cough, dyspnea, generalized weakness, and supplemental oxygen requirement persisted. An extensive infectious evaluation was performed. Blood cultures remained negative. Respiratory viral testing and testing for *Legionella*, *Streptococcus pneumoniae*, *Mycoplasma*, *Chlamydia*, and *Coccidioides* were unrevealing. The absence of persistent fever or leukocytosis, low procalcitonin, negative blood cultures, and lack of clinical improvement despite antimicrobial therapy made conventional bacterial pneumonia less likely but did not exclude localized, atypical, or partially treated infection.

Pulmonology was consulted on HD 4 for persistent hypoxic respiratory failure and diffuse bilateral GGOs. At that time, the differential diagnosis was broadened beyond infection to include atypical infection, OP, inflammatory interstitial lung disease, hypersensitivity pneumonitis, eosinophilic lung disease, and an autoimmune or vasculitic pulmonary process. Additional fungal and atypical infectious studies were obtained, and autoimmune evaluation, including ANCA testing, was recommended. Antibiotics were subsequently discontinued when the clinical picture was considered increasingly inconsistent with bacterial pneumonia.

The patient continued to require approximately 1-2 L/min of supplemental oxygen. Given his history of heart failure and a recent cardiac catheterization at an outside hospital for evaluation of suspected coronary artery disease, during which no stents were placed, cardiogenic pulmonary edema remained a competing consideration. Transthoracic echocardiography demonstrated an ejection fraction of approximately 45% with mild diastolic dysfunction. Heart-failure-directed medical therapy, including metoprolol succinate 50 mg daily, dapagliflozin 10 mg daily, and sacubitril/valsartan 24/26 mg twice daily, was initiated on HD 6. Repeat CT of the chest on HD 12 demonstrated persistent diffuse, basilar-predominant bilateral GGOs with an interval increase in the consolidative component at the left lung base and focal left lower lobe bronchiectasis (Figure 1B). No significant pleural effusion or mediastinal lymphadenopathy was present. Mild cardiomegaly and pulmonary vascular congestion were also noted. Given the persistence of his symptoms and radiographic abnormalities, evaluation for an additional pulmonary process was continued.

The persistence of pulmonary abnormalities despite antimicrobial therapy, together with an unrevealing infectious evaluation, increased concern for a non-infectious pulmonary process. The differential diagnosis was broadened to include OP, eosinophilic lung disease, hypersensitivity pneumonitis, drug-related pulmonary disease, and pulmonary involvement from systemic vasculitis. Bronchoscopy with BAL was subsequently performed for further evaluation. The procedure demonstrated thin secretions throughout the examined airways without active endobronchial bleeding. BAL demonstrated a mixed inflammatory cellular profile with 41% polymorphonuclear leukocytes, 23% lymphocytes, 16% macrophages, 13% eosinophils, and 7% bronchial epithelial cells (Table 1). Cytologic examination demonstrated acute inflammation with eosinophils and reactive bronchial epithelial cells without evidence of malignancy.

The patient's earlier hemoptysis had resolved before bronchoscopy. The BAL fluid was described as cloudy and white, without progressively bloody aliquots or active bleeding. A quantitative BAL red blood cell count was not documented. Hemosiderin-laden macrophages and iron staining were not documented. Therefore, the available bronchoscopy findings did not support diffuse alveolar hemorrhage at the time of the procedure, although prior or resolving diffuse alveolar hemorrhage could not be definitively excluded.

**Table 1:** Bronchoalveolar lavage (BAL) findings. BAL demonstrated a mixed inflammatory cellular profile with elevated polymorphonuclear leukocytes (41%) and eosinophils (13%) and decreased macrophages (16%). Cytologic examination demonstrated acute inflammation with eosinophils and reactive bronchial epithelial cells without evidence of malignancy. Although HSV-1 DNA and bacterial and *Candida* species were detected in BAL specimens, subsequent Infectious Disease evaluation considered these findings more consistent with colonization, contamination, or viral reactivation than active pulmonary infection. *Pneumocystis* testing and fungal studies were negative. AFB fluorochrome staining demonstrated no acid-fast bacilli (AFB), with no AFB identified on culture at the time of follow-up. AFB: acid-fast bacilli; BAL: bronchoalveolar lavage; DFA: direct fluorescent antibody; HSV-1: herpes simplex virus type 1; HSV-2: herpes simplex virus type 2; N/A: not applicable; PCR: polymerase chain reaction.

BAL finding	Result	Reference range
Polymorphonuclear leukocytes	41%	2-11%
Lymphocytes	23%	2-25%
Macrophages	16%	60-80%
Eosinophils	13%	<1%
Bronchial epithelial cells	7%	3-21%
Cytology	Acute inflammation with eosinophils	N/A
Malignant cells	None identified	None identified
Pneumocystis DFA	Not detected	Not detected
AFB fluorochrome stain	No AFB identified	No AFB identified
AFB culture	No AFB to date on follow-up	No growth
Final fungal culture	No growth of yeast/fungi	No growth
HSV-1 PCR	Detected	Not detected
HSV-2 PCR	Not detected	Not detected

Cytologic examination of the BAL demonstrated acute inflammation with eosinophils and reactive bronchial epithelial cells without malignant cells. Rare fungal hyphae and pseudohyphae morphologically consistent with *Candida* species were identified on special staining. The pathology report noted that these findings most likely represented oropharyngeal contamination, although active infection could not be excluded solely on morphologic grounds.

The presence of 13% eosinophils in BAL fluid was abnormal and supported a pulmonary eosinophilic inflammatory component. However, the degree of BAL eosinophilia was below the levels typically used to support a specific diagnosis of acute or chronic eosinophilic pneumonia. Accordingly, the BAL was interpreted as evidence of eosinophilic inflammation rather than diagnostic of a particular eosinophilic pneumonia syndrome. Microbiologic testing from BAL added complexity to the case. Respiratory cultures demonstrated methicillin-resistant *Staphylococcus aureus* (MRSA) and *Escherichia coli*, *Candida* species were identified cytologically, and herpes simplex virus type 1 (HSV-1) DNA was detected by PCR. MRSA growth was reported approximately 72 hours after BAL collection.

Because these findings raised concern for possible ongoing infection, Infectious Disease was consulted on HD 20. The positive BAL results were interpreted in the context of the patient's overall clinical course. He had a subacute illness without ongoing fever or leukocytosis, had not responded as expected to broad-spectrum antimicrobial therapy, and had undergone an otherwise extensive negative infectious evaluation. There was no convincing clinical syndrome of invasive candidiasis or HSV pneumonitis. Infectious Disease therefore concluded that the bacterial growth most likely reflected airway colonization, the *Candida* represented oropharyngeal contamination, and the HSV-1 PCR positivity likely reflected contamination or viral reactivation rather than invasive pulmonary infection. Vancomycin and other antimicrobial therapies were discontinued.

Additional infectious testing further reduced concern for an occult infectious cause. *Pneumocystis jirovecii* direct fluorescent antibody (DFA) testing was negative. Strongyloides IgG was negative. HIV testing was negative. The final fungal culture from the BAL showed no growth of yeast or mold. Acid-fast bacilli (AFB) fluorochrome staining demonstrated no AFB, and follow-up culture continued to show no AFB at the time of review. These negative studies reduced concern for the specific infections tested but did not exclude all infectious causes. When interpreted together with the patient's clinical course, lack of sustained response to antimicrobial therapy, and Infectious Disease assessment, they contributed to greater consideration of a non-infectious inflammatory pulmonary process.

Further laboratory evaluation demonstrated peripheral eosinophilia. The absolute eosinophil count reached a peak of 1.695 × 10³/µL, with a relative eosinophil count of 15%, on HD 8. Immediately before corticosteroid initiation on HD 19, the absolute eosinophil count was 0.79 × 10³/µL (10%). Total IgE was elevated at 771 kU/L (reference range: ≤114 kU/L). Because an allergic or environmental pulmonary process remained in the differential, a Southwest environmental allergy panel was obtained. Specific IgE testing for *Alternaria*, *Cladosporium*, *Dermatophagoides farinae*, Bermuda grass, perennial rye grass, cat and dog dander, mugwort, oak, and western ragweed was negative. ANCA screening by indirect immunofluorescence was repeatedly positive. The pattern was specifically reported as atypical p-ANCA, with a titer of 1:40 followed by an increase to 1:80. However, antigen-specific testing demonstrated MPO antibody <1.0 AI and PR3 antibody <1.0 AI on both evaluations, indicating no detectable MPO-ANCA or PR3-ANCA (Table 2).

**Table 2:** Major diagnostic findings contributing to the evaluation of persistent bilateral pulmonary opacities. BAL eosinophilia, peripheral eosinophilia, and elevated total IgE supported an eosinophilic inflammatory phenotype but were not diagnostic of a specific eosinophilic lung disorder. Repeated atypical p-ANCA positivity by indirect immunofluorescence, with titers increasing from 1:40 to 1:80, raised consideration of systemic vasculitis; however, antigen-specific MPO and PR3 antibodies remained negative, and the ANCA findings alone were not diagnostic of AAV. Infectious studies, together with the lack of clinical improvement with antimicrobial therapy and Infectious Disease assessment, reduced concern for the specific infections evaluated but did not exclude all infectious causes. * Procalcitonin values of 0.04-0.06 ng/mL were low. These values did not support systemic bacterial infection and were not interpreted as independently excluding localized pulmonary infection or sepsis. † Reference range for the tested environmental-specific-IgE assays. AAV: ANCA-associated vasculitis; AEC: absolute eosinophil count; AFB: acid-fast bacilli; AI: antibody index; ANCA: antineutrophil cytoplasmic antibody; BAL: bronchoalveolar lavage; CT: computed tomography; DFA: direct fluorescent antibody; GGO: ground-glass opacity; IgE: immunoglobulin E; IIF: indirect immunofluorescence; MPO: myeloperoxidase; N/A: not applicable; p-ANCA: perinuclear antineutrophil cytoplasmic antibody; PR3: proteinase 3.

Finding	Result	Reference range	Clinical interpretation
Initial CT chest	Diffuse bilateral GGOs	N/A	Infection, edema, inflammatory lung disease, or vasculitis considered
Antibiotic response	Persistent symptoms/infiltrates	N/A	Reduced likelihood of conventional bacterial pneumonia; did not exclude localized, atypical, or partially treated infection
Procalcitonin	0.04-0.06 ng/mL	≤0.5 ng/mL*	Low procalcitonin; did not support systemic bacterial infection but did not exclude localized infection
BAL eosinophils	13%	<1%	Demonstrated BAL eosinophilia consistent with an eosinophilic inflammatory component, but not specific for eosinophilic pneumonia
BAL cytology	Acute inflammation with eosinophils	N/A	Supported an eosinophilic inflammatory component
Peripheral eosinophilia	Peak AEC 1.695 × 10³/µL; peak eosinophils 15%	AEC 0.00-0.78 × 10³/µL; eosinophils 0-7%	Supported the presence of peripheral eosinophilia; this finding is non-specific
Total IgE	771 kU/L	≤114 kU/L	Elevated; non-specific finding
ANCA	Atypical p-ANCA positive by IIF, 1:40 → 1:80; MPO <1.0 AI and PR3 <1.0 AI on both evaluations	Atypical p-ANCA <1:20; MPO/PR3 <1.0; AI = no antibody detected	Persistent atypical p-ANCA positivity by IIF with negative antigen-specific MPO/PR3; not diagnostic of AAV
Strongyloides IgG	Negative	Negative	Reduced concern for *Strongyloides* infection before immunosuppression
Pneumocystis DFA	Not detected	Not detected	Reduced concern for *Pneumocystis* infection
Final fungal culture	No growth of yeast/fungi	No growth	Reduced concern for fungal infection on culture
AFB fluorochrome stain	No AFB identified	No AFB identified	No AFB identified on smear
AFB culture	No AFB to date	No growth	No AFB identified on follow-up culture at the time of review
Environmental allergen panel	Negative	<0.10 kU/L†	No sensitization to tested environmental allergens
Serum creatinine	Approximately 0.5-0.7 mg/dL	0.5-1.5 mg/dL	Preserved renal function
Neuropathic symptoms	Chronic lower-extremity neuropathy, most pronounced in the feet; reportedly worsened during the pulmonary illness	N/A	Considered most consistent with diabetic neuropathy; no inpatient neurologic evaluation or electrodiagnostic testing was performed

The combination of persistent pulmonary infiltrates, peripheral eosinophilia, BAL eosinophilia, elevated IgE, constitutional symptoms, and neuropathic complaints raised concern for an eosinophilic systemic inflammatory disorder. The abnormal ANCA screen additionally raised the possibility of AAV. Pulmonology considered EGPA and microscopic polyangiitis (MPA), in addition to OP and eosinophilic lung disease.

However, several findings argued against assigning a definitive AAV diagnosis during the hospitalization. The ANCA pattern was atypical rather than a classic MPO- or PR3-associated pattern, both antigen-specific assays were negative, renal function remained preserved with serum creatinine approximately 0.5-0.7 mg/dL (reference range: 0.5-1.5 mg/dL), and tissue confirmation of vasculitis was not available. The patient had no documented history of asthma or inhaler use, and no wheezing was documented during the hospitalization. No nasal polyps were identified on bilateral inspection of the nares; however, formal otolaryngologic evaluation and sinus imaging were not performed. His chronic neuropathy predominantly involved the lower extremities and was most pronounced in the feet. Although the neuropathy was considered most consistent with diabetic neuropathy and predated the current pulmonary illness, the patient reported worsening during the period in which his pulmonary symptoms developed. Neurology did not evaluate him during the hospitalization, and electrodiagnostic testing was not available. Therefore, EGPA and other forms of AAV remained diagnostic considerations rather than confirmed diagnoses.

The patient had a history of eczema treated with dupilumab. Dupilumab was held during the hospitalization because of concern that it could be contributing to or complicating the eosinophilic pulmonary process. The available documentation did not establish a sufficiently clear temporal relationship between dupilumab exposure and the onset of the pulmonary illness to attribute the syndrome to dupilumab.

Prednisone 60 mg orally daily (approximately 1 mg/kg/day based on a documented weight of 64.5 kg) was initiated on HD 19 for the suspected inflammatory pulmonary process. No intravenous corticosteroids were administered. On HD 19, the patient remained on 2 L/min of supplemental oxygen with an oxygen saturation of 98%. On HD 20, he remained on 2 L/min with an oxygen saturation of 98%. By HD 21, he was on room air with an oxygen saturation of 98%, and on HD 22, he remained on room air with an oxygen saturation of 97%. The absolute eosinophil count decreased from 0.79 × 10³/µL (10%) immediately before corticosteroid initiation on HD 19 to 0.00 × 10³/µL (0%) on HD 20.

The temporal improvement following prednisone was compatible with an inflammatory component but was not specific for a steroid-responsive eosinophilic disorder. Heart-failure-directed medical therapy had been initiated on HD 6, approximately two weeks before prednisone, and there was no documented change in fluid balance at the time corticosteroids were initiated. Nevertheless, cardiogenic pulmonary edema remained a potential contributor to the pulmonary abnormalities and subsequent improvement; therefore, the clinical and imaging improvement could not be attributed solely to corticosteroid therapy.

Follow-up CT of the chest on HD 20 demonstrated interval improvement in the basilar-predominant GGOs with decreased interlobular septal thickening and reticulation (Figure 1C). New right lower lobe tree-in-bud nodular opacities were also identified. Importantly, the radiologist favored improving pulmonary edema secondary to congestive heart failure as an explanation for at least part of the interval radiographic improvement. Therefore, although the patient's clinical course was compatible with an inflammatory pulmonary process, the radiographic improvement could not be attributed exclusively to corticosteroid therapy. Heart-failure-directed medical therapy had been initiated on HD 6, although sacubitril/valsartan was subsequently held during portions of the hospitalization in the setting of persistent hyponatremia.

Hyponatremia represented an additional complication during the prolonged hospitalization, with serum sodium ranging from approximately 125 to 134 mmol/L (reference range: 135-145 mmol/L). Nephrology evaluation demonstrated findings considered consistent with the syndrome of inappropriate antidiuretic hormone secretion (SIADH), potentially related to the pulmonary process and compounded by poor oral intake and medication effects. Management included fluid restriction of 1 L/day, salt tablets, urea therapy, and a brief normal saline infusion. Serum sodium ultimately improved to 135 mmol/L.

Although his oxygenation and respiratory symptoms improved, he continued to report residual generalized weakness, pruritus, and neuropathic symptoms. At discharge on HD 27, he remained on room air with an oxygen saturation of 98%. Prednisone 60 mg orally daily was continued, with a documented plan to complete four weeks at 60 mg daily followed by tapering by 5 mg weekly. Trimethoprim-sulfamethoxazole was prescribed for *Pneumocystis* prophylaxis during high-dose corticosteroid therapy. He was discharged to a skilled nursing facility with outpatient pulmonology and rheumatology follow-up. Repeat CT imaging and ANCA serologies were planned, with consideration of tissue biopsy if pulmonary abnormalities failed to resolve or systemic manifestations progressed.

## Discussion

This case illustrates the difficulty of evaluating persistent bilateral pulmonary opacities when several competing processes are present. The patient initially had a reasonable working diagnosis of multifocal pneumonia: he reported fever, had bilateral infiltrates and hypoxemia, and had recently been hospitalized for pneumonia. What changed the diagnostic trajectory was his failure to improve despite multiple courses of antibiotics.

Non-resolving pulmonary infiltrates have a broad differential that includes inadequately treated infection, OP, eosinophilic pneumonia, pulmonary edema, diffuse alveolar hemorrhage, hypersensitivity pneumonitis, drug-induced lung injury, malignancy, and autoimmune pulmonary disease. In this case, the absence of ongoing fever or leukocytosis, low procalcitonin, extensive negative infectious testing, and lack of response to broad-spectrum antibiotics made conventional bacterial pneumonia progressively less likely.

The BAL results were particularly helpful, although they initially complicated the picture. MRSA, *E. coli*, *Candida*, and HSV-1 were detected, findings that could have led to prolonged antimicrobial treatment. Infectious Disease interpreted these results in the context of the patient's clinical course and considered them unlikely to represent active pulmonary infection. Negative *Pneumocystis*, *Strongyloides*, fungal, and AFB studies further reduced concern for an occult infectious process.

More informative was the BAL cellular differential. Eosinophils normally account for less than 1% of BAL cells [3], whereas this patient's BAL contained 13% eosinophils, and cytology independently demonstrated eosinophilic inflammation. This was clearly abnormal but below the BAL eosinophil levels typically associated with classic eosinophilic pneumonia. Acute eosinophilic pneumonia is generally associated with BAL eosinophils of at least 25%, whereas chronic eosinophilic pneumonia commonly demonstrates BAL eosinophilia of 40% or greater [3]. The 13% BAL eosinophilia in this case was therefore interpreted as evidence of an eosinophilic inflammatory component rather than diagnostic of either acute or chronic eosinophilic pneumonia.

Peripheral eosinophilia and elevated IgE strengthened the impression of an eosinophilic inflammatory process, but neither finding is disease-specific. Parasitic infection, allergic disease, medication reactions, eosinophilic pneumonia, hypereosinophilic disorders, and EGPA can produce a similar laboratory profile. The negative *Strongyloides* serology was particularly relevant before initiating corticosteroids, while the negative environmental allergy panel did not identify sensitization to the allergens tested.

EGPA was considered because of the combination of peripheral and BAL eosinophilia, pulmonary infiltrates, neuropathic symptoms, and ANCA positivity. The patient's peak absolute eosinophil count was 1.695 × 10³/µL, with a relative eosinophil count of 15%. However, several features limited diagnostic certainty. He had no documented history of asthma or inhaler use, and no wheezing was documented during the hospitalization. No nasal polyps were identified on bilateral inspection of the nares, although formal otolaryngologic evaluation and sinus imaging were not performed. The ANCA pattern was atypical p-ANCA by indirect immunofluorescence, while antigen-specific MPO and PR3 antibodies remained negative. In addition, his chronic neuropathy predominantly involved the lower extremities and was considered most consistent with diabetic neuropathy. Although the patient reported worsening of his neuropathic symptoms during the pulmonary illness, the neuropathy predated the current presentation, and neither inpatient neurologic evaluation nor electrodiagnostic testing was performed. No tissue biopsy demonstrated eosinophilic or necrotizing vasculitis. Therefore, EGPA remained a diagnostic consideration rather than a confirmed diagnosis.

The 2022 ACR/EULAR EGPA criteria are classification criteria intended for application after a diagnosis of small- or medium-vessel vasculitis has been established and mimics have been excluded [4]. Accordingly, individual features in this patient should not be used to establish EGPA solely by calculating a classification score.

Indirect immunofluorescence was repeatedly positive, with an atypical p-ANCA titer increasing from 1:40 to 1:80. However, antigen-specific MPO and PR3 antibodies remained negative. Therefore, the immunofluorescence positivity did not establish AAV. Current recommendations place greater diagnostic weight on antigen-specific MPO-ANCA and PR3-ANCA assays than on immunofluorescence patterns alone [5]. Atypical p-ANCA is less specific for AAV and can occur in other inflammatory and autoimmune conditions. Negative MPO and PR3 antibodies do not completely exclude vasculitis; however, isolated atypical p-ANCA positivity should not be considered diagnostic of AAV.

MPA was also considered because of the pulmonary abnormalities and ANCA positivity. However, the atypical p-ANCA pattern was not accompanied by MPO antibodies, renal function remained preserved, and there was no established glomerulonephritis or pulmonary-renal syndrome. The patient's earlier hemoptysis had resolved before bronchoscopy, and the BAL was not progressively bloody. A quantitative BAL red blood cell count, hemosiderin-laden macrophages, and iron staining were not documented. In diffuse alveolar hemorrhage, progressively hemorrhagic BAL aliquots and evidence of alveolar bleeding can support the diagnosis; however, the absence of these findings at a later bronchoscopy does not definitively exclude prior or resolving hemorrhage [6]. Thus, the available findings did not support active diffuse alveolar hemorrhage at the time of bronchoscopy, although prior or resolving hemorrhage could not be definitively excluded. Taken together, these findings made a definitive diagnosis of MPA less compelling.

OP was another important consideration given the persistent bilateral pulmonary opacities, lack of sustained response to antimicrobial therapy, and subsequent clinical improvement during corticosteroid treatment. However, tissue confirmation was not obtained, and cardiogenic pulmonary edema remained an important competing explanation for the imaging abnormalities and their subsequent improvement. Therefore, OP remained a diagnostic consideration rather than a histologically confirmed diagnosis.

Dupilumab exposure was also considered because peripheral eosinophilia, eosinophilic pneumonia, and cases of EGPA have been reported during dupilumab therapy [7-10]. However, the available clinical history did not establish a clear temporal relationship between dupilumab initiation and the current pulmonary illness. Dupilumab was therefore held, but causality could not be established.

Heart failure was an important confounder. The patient had an ejection fraction of approximately 45%, cardiomegaly, mild diastolic dysfunction, and pulmonary vascular congestion. Heart-failure-directed medical therapy had been initiated on HD 6, approximately two weeks before prednisone, and there was no documented change in fluid balance at the time corticosteroids were initiated. On follow-up CT, the radiologist specifically favored improving pulmonary edema as an explanation for at least part of the interval improvement in the GGOs. Therefore, cardiogenic pulmonary edema likely contributed to the pulmonary abnormalities and their subsequent improvement, limiting the ability to attribute the radiographic response specifically to prednisone.

At the same time, the eosinophilic and immunologic findings raised concern for a concurrent inflammatory process that was not fully explained by cardiogenic pulmonary edema alone. BAL and peripheral eosinophilia, eosinophils on cytology, elevated IgE, neuropathic symptoms, and atypical p-ANCA positivity supported consideration of an eosinophilic inflammatory process. However, these findings were individually non-specific and did not establish a single unifying diagnosis. A multifactorial process was therefore considered, with cardiogenic pulmonary edema potentially contributing to the imaging abnormalities alongside a separate eosinophilic inflammatory process.

The temporal improvement following prednisone was compatible with an inflammatory component but was not specific for a steroid-responsive eosinophilic disorder. OP, eosinophilic lung disease, medication-associated pulmonary inflammation, and pulmonary manifestations of systemic autoimmune disease may all improve with corticosteroid therapy. In this patient, the absolute eosinophil count decreased from 0.79 × 10³/µL (10%) immediately before prednisone on HD 19 to 0.00 × 10³/µL (0%) on HD 20, while supplemental oxygen was discontinued by HD 21. Although this temporal relationship supported the possibility of a corticosteroid-responsive inflammatory component, concurrent heart-failure treatment and radiographic evidence of improving pulmonary edema precluded attribution of the overall clinical and imaging improvement solely to corticosteroid therapy.

The most appropriate characterization of this case is an eosinophilic inflammatory pulmonary process with atypical p-ANCA positivity and concern for, but not confirmation of, AAV. The value of this case is not that atypical p-ANCA positivity established vasculitis, but that the combination of non-resolving pulmonary infiltrates, eosinophilic inflammation, systemic symptoms, and abnormal serology appropriately prompted evaluation for pulmonary vasculitis while illustrating the limitations of serologic findings in the absence of a compatible clinical phenotype or tissue confirmation. The major diagnostic and therapeutic events during the hospitalization are summarized in Table 3.

**Table 3:** Clinical timeline of diagnostic evaluation and management during hospitalization. Summary of the patient’s clinical course from the period preceding admission through discharge. The timeline highlights the initial treatment for presumed multifocal pneumonia, persistence of bilateral GGOs despite antimicrobial therapy, subsequent bronchoscopy demonstrating BAL eosinophilia, and an infectious evaluation that reduced concern for the specific infections tested without excluding all infectious causes. Repeated atypical p-ANCA positivity with negative MPO and PR3 antibodies raised concern for possible AAV but was not diagnostic. Prednisone was initiated on HD 19 for the suspected inflammatory pulmonary process, followed by suppression of peripheral eosinophilia and improvement in oxygen requirement. Concurrent heart-failure-directed therapy and possible cardiogenic pulmonary edema limited attribution of the clinical and radiographic improvement solely to corticosteroid therapy. Outpatient pulmonology and rheumatology follow-up were planned. AEC: absolute eosinophil count; AFB: acid-fast bacilli; ANCA: antineutrophil cytoplasmic antibody; BAL: bronchoalveolar lavage; CT: computed tomography; GGO: ground-glass opacity; HD: hospital day; HSV-1: herpes simplex virus type 1; IgE: immunoglobulin E; MPO: myeloperoxidase; MRSA: methicillin-resistant *Staphylococcus aureus*; p-ANCA: perinuclear antineutrophil cytoplasmic antibody; PR3: proteinase 3; SIADH: syndrome of inappropriate antidiuretic hormone secretion; OP: organizing pneumonia; AAV: ANCA-associated vasculitis.

Hospital day	Clinical course, investigations, and management
Prior to admission	Recently hospitalized and treated for presumed pneumonia with antibiotics, including amoxicillin, without significant improvement. Developed progressive weakness, fatigue, dry cough, dyspnea, dizziness, and reported fever.
HD 1-2	Presented with persistent respiratory and constitutional symptoms requiring approximately 2 L/min of supplemental oxygen. Chest radiograph demonstrated cardiomegaly with bilateral multifocal infiltrates, and CT pulmonary angiography on HD 2 demonstrated diffuse bilateral GGOs without pulmonary embolism. Mild hyponatremia, absence of significant leukocytosis, and low procalcitonin were noted. Cefepime and azithromycin were administered empirically from HD 2 through HD 4 for presumed multifocal pneumonia.
HD 4	Pulmonology was consulted for persistent hypoxic respiratory failure and bilateral GGOs despite antimicrobial therapy. The differential diagnosis broadened to include atypical infection, OP, inflammatory interstitial lung disease, hypersensitivity pneumonitis, eosinophilic lung disease, and an autoimmune or vasculitic pulmonary process. Additional infectious and autoimmune testing, including ANCA, was pursued.
HD 6	Heart-failure-directed medical therapy with metoprolol succinate, dapagliflozin, and sacubitril/valsartan was initiated. Cardiogenic pulmonary edema remained a competing explanation for the pulmonary abnormalities.
HD 8	Peripheral eosinophilia reached a peak absolute eosinophil count of 1.695 × 10³/µL (15%).
HD 12	Repeat CT demonstrated persistent basilar-predominant bilateral GGOs with increased left basilar consolidation. Mild cardiomegaly and pulmonary vascular congestion were also present. The differential included OP, eosinophilic lung disease, hypersensitivity pneumonitis, drug-related pulmonary disease, and pulmonary vasculitis.
During hospitalization	Bronchoscopy with BAL demonstrated thin secretions without active endobronchial bleeding. BAL showed a mixed inflammatory profile with 41% neutrophils, 23% lymphocytes, 16% macrophages, and 13% eosinophils. Cytology demonstrated acute inflammation with eosinophils without malignant cells. The BAL was not progressively bloody; a quantitative BAL red blood cell count, hemosiderin-laden macrophages, and iron staining were not documented.
During hospitalization	Autoimmune evaluation demonstrated repeatedly positive atypical p-ANCA by indirect immunofluorescence, with titers increasing from 1:40 to 1:80, while MPO and PR3 antibodies remained negative. Total IgE was elevated at 771 kU/L.
After BAL	BAL testing detected HSV-1 PCR, MRSA, *Escherichia coli*, and *Candida* species. MRSA growth was reported approximately 72 hours after BAL collection. *Pneumocystis* testing and *Strongyloides* serology were negative. AFB staining demonstrated no AFB, fungal culture showed no growth, and no AFB had been identified on culture at the time of follow-up. Infectious Disease considered the detected organisms more consistent with colonization, contamination, or viral reactivation than active pulmonary infection.
HD 19	Prednisone 60 mg orally daily (approximately 1 mg/kg/day based on a weight of 64.5 kg) was initiated for the suspected inflammatory pulmonary process; no intravenous corticosteroids were administered. Immediately before prednisone, the AEC was 0.79 × 10³/µL (10%). The patient remained on 2 L/min of supplemental oxygen with an oxygen saturation of 98%. Dupilumab was held.
HD 20	AEC decreased to 0.00 × 10³/µL (0%). The patient remained on 2 L/min of supplemental oxygen with an oxygen saturation of 98%. Follow-up CT demonstrated interval improvement in the basilar-predominant GGOs and interlobular septal thickening, with new right lower lobe tree-in-bud opacities. The radiologist favored improving pulmonary edema as an explanation for at least part of the radiographic improvement.
HD 21-22	The patient was on room air with oxygen saturations of 98% on HD 21 and 97% on HD 22. The temporal response was compatible with an inflammatory component but was not diagnostically specific because heart-failure treatment preceded corticosteroid initiation and pulmonary edema remained a potential contributor.
Late hospitalization	Weakness, pruritus, and neuropathic symptoms persisted. Hyponatremia attributed primarily to SIADH improved with fluid restriction of 1 L/day, salt tablets, urea therapy, and a brief normal saline infusion. Renal function remained preserved.
HD 27/Discharge	The patient remained on room air with an oxygen saturation of 98% and was discharged to a skilled nursing facility. Prednisone 60 mg orally daily was continued, with a documented plan to complete four weeks at 60 mg daily followed by tapering by 5 mg weekly. Trimethoprim-sulfamethoxazole was prescribed for *Pneumocystis* prophylaxis. Outpatient pulmonology and rheumatology follow-up were planned.

## Conclusions

This case highlights the diagnostic challenge of persistent pulmonary infiltrates that fail to improve with antimicrobial therapy. The combination of peripheral and BAL eosinophilia, systemic symptoms, and atypical p-ANCA positivity raised concern for an eosinophilic inflammatory pulmonary process and possible AAV, including EGPA. However, atypical p-ANCA positivity with negative MPO and PR3 antibodies is not diagnostic of AAV, and no tissue confirmation of vasculitis was obtained. The temporal improvement in peripheral eosinophilia and oxygen requirement following prednisone was compatible with an inflammatory component but was not diagnostically specific, particularly in the setting of heart-failure-directed therapy and possible cardiogenic pulmonary edema. This case emphasizes the importance of broadening the differential diagnosis in non-resolving pneumonia while avoiding diagnostic anchoring on non-specific ANCA results and recognizing that multiple pulmonary processes may coexist.
